# A Combat Journey of Rehabilitation in Pre- and Post-chemotherapy for Esophagus Carcinoma

**DOI:** 10.7759/cureus.58202

**Published:** 2024-04-13

**Authors:** Aditi Akhuj, Vrushali Athawale, Tejaswini Fating

**Affiliations:** 1 Oncology Physiotherapy, Ravi Nair Physiotherapy College, Datta Meghe Institute of Higher Education & Research, Wardha, IND; 2 Community Health Physiotherapy, Ravi Nair Physiotherapy College, Datta Meghe Institute of Higher Education & Research, Wardha, IND

**Keywords:** esophageal adenocarcinoma, rehabilitation, physiotherapy, persistent dysphagia, squamous cell carcinoma, esophageal cancer

## Abstract

Esophageal cancer is a malignant epithelial alteration that takes place in the middle or upper part of the esophagus. Given the escalating population of individuals who have successfully overcome esophageal cancer, the significance of addressing disease- and treatment-associated complaints and symptoms is increasingly pertinent. This highlights the necessity of interventions meant to enhance quality of life (QOL). We present the case of a 60-year-old female diagnosed with esophageal squamous cell carcinoma who presented with chief complaints of generalized weakness, breathlessness, and nausea. Patient-tailored physiotherapy pre-rehabilitation and post-rehabilitation, including strengthening exercises, breathing exercises, dyspnea-relieving positions, Mendelsohn maneuver, Shaker exercise, among others, were administered. These interventions proved effective in enhancing the patient’s functional independence and QOL. Treatment commenced one week prior to the first chemotherapy session. Post-chemotherapy intervention was provided, and on the day of discharge, follow-up revealed improved strength and QOL.

## Introduction

Esophageal cancer ranks as the ninth most prevalent malignancy globally and stands as the sixth leading cause of cancer-related mortality on a global scale [1]. Esophageal squamous cell carcinoma (ESCC) constitutes 90% of the total incidence of esophageal cancer cases. It is a malignant epithelial alteration that takes place in the middle or upper part of the esophagus [2]. Esophageal carcinoma manifests in two primary histological types, namely adenocarcinoma and squamous cell carcinoma, with the latter being the predominant histological subtype worldwide [3]. Males are more likely than females to have either of these histological classifications. The rising number of ESCC cases being diagnosed at an early stage of the disease is partly responsible for the better five-year survival rate. Patients with esophageal cancer who were diagnosed at the localized and regional stages showed a 21.1% increase in their overall five-year survival rate. Risk factors for esophageal cancer include Barrett’s esophagus, smoking, poor oral health, human papillomavirus, hot tea drinking, low socioeconomic status, and inadequate intake of fresh fruits and vegetables [4]. The onset of menopause represents an independent risk factor for esophageal cancer [5].

Conversely, the utilization of hormonal therapy, comprising estrogen and progesterone, is correlated with a reduced risk of ESCC in postmenopausal women [6]. The most common side effects of esophageal cancer and its therapies are weight loss, malnutrition, and sarcopenia, which lead to severe impairments in physical functioning and quality of life (QOL) from the time of diagnosis until survivorship [7]. Historically, esophagectomy has been the preferred therapeutic approach for localized, non-invasive esophageal tumors. Treatment for more severe, incurable diseases is palliative and focuses on regaining swallowing and feeding abilities; gastrostomy tubes are frequently necessary to maintain sufficient nourishment. In certain instances, a combination of chemoradiation is employed to decelerate tumor progression and extend the duration before the initiation of palliative interventions becomes necessary [8]. Chemotherapy effectively decreases the disease burden in cases of esophageal cancer and has a number of beneficial effects; it leads to a reduction in the size of the tumor, contributes to the control or elimination of cancerous cells, alleviates symptoms associated with esophageal cancer, such as difficulty swallowing, and can extend survival rates [9].

Esophageal cancer represents a significant malignancy typically managed through multimodal interventions and intricate surgical resection procedures. As therapeutic intervention transition centers focus on advanced recovery methodologies, the responsibilities of physiotherapists have broadened. Previous research has demonstrated the substantial positive effects of fitness programs started after cancer treatment on QOL. There exists a robust foundation supporting customized physiotherapy interventions in the care and treatment of individuals diagnosed with esophageal cancer. It aids in the coping of cancer patients with symptoms and treatment. The goal of pre-rehabilitation programs is to maximize patients’ outcomes both before and after undergoing surgery for cancer. Prior studies involving surgical patients reveal that pre-rehabilitation enhances pre-operative physical fitness and mitigates the adverse effects of neoadjuvant chemotherapy on fitness. Chemotherapy has an impact on the patient’s overall QOL and affects several systems, including the digestive, hematopoietic, and cardiovascular systems [10].

The term “oropharyngeal dysphagia” precisely describes the unsafe or ineffective passage of liquid or solid from the mouth into the esophagus [11]. The Mendelsohn maneuver may be contemplated as a rehabilitative intervention involving strength enhancement, skill training, or relearning techniques for the management of dysphagia. It aims to lengthen the cricopharyngeal aperture to facilitate bolus passage into the esophagus and to better elevate the larynx for airway protection [12]. The Shaker exercise is an exercise regimen designed to strengthen the suprahyoid muscles. It consists of both isometric and isokinetic components. Exercise has been demonstrated to increase the anterior excursion of the hyolaryngeal complex during swallowing, improve the traction force, one of the opening mechanisms of the upper esophageal sphincter, activate the contraction of the thyrohyoid ligament, and strengthen the suprahyoid muscle group [13]. This is a case report of a 60-year-old female with ESCC, and the goal of this paper was to prove the importance of physiotherapy in this condition.

## Case presentation

Patient information

A 60-year-old female presented with a four-month history of progressive dysphagia, breathlessness, generalized weakness, and pain in the right lower chest wall, which was gradual in onset and throbbing in nature for the past 20 days. The patient reported no history of smoking or secondhand smoke exposure, tobacco chewing, or alcohol consumption. She had no family history of chronic illness or cancer. She presented with a persistent loss of appetite over the preceding months and confirmed a reduction in body weight within the last month. The patient had trouble swallowing solids and semi-solid foods. Upon physical examination, no abnormalities were identified. Chemotherapy was suggested (10 cycles), and the patient was referred to the oncology physiotherapy department for fitness and preventing post-chemotherapy complications.

Clinical findings

The patient provided both written and verbal consent prior to the commencement of the physical examination. She was conscious, cooperative, and well-oriented to person, place, and time. The patient was afebrile and hemodynamically stable. The patient was seen in a supine lying posture with the head end elevated to 30 degrees. She was ectomorphic, with a body mass index of 18 kg/m^2^. On observation, bilateral supraclavicular retraction was noted. On head and neck examination, the water swallowing test demonstrated an inability to swallow with choking and/or breathing changes. On musculoskeletal examination, upper and lower limb strength was 3 out of 5 on both sides (3: full ROM against gravity; 5: full ROM against gravity), maximal resistance, and modified Medical Research Council (mMRC) grading was grade 2, which was “On level ground, I walk slower than people of the same age because of breathlessness or have to stop for breath when walking at my own pace.” On auscultation, air entry was reduced bilaterally.

Diagnostic assessment

**Figure 1:**
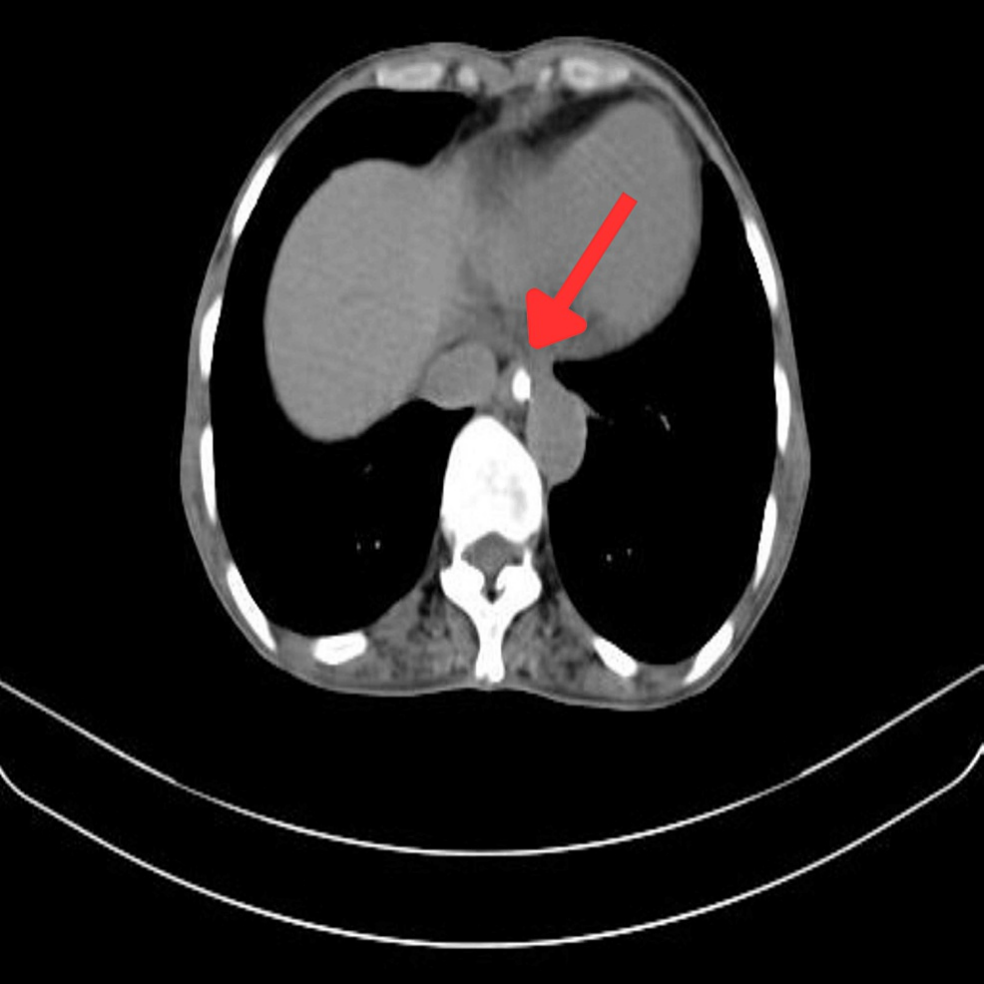
CECT thorax with the abdomen The red arrow points at the asymmetrical circumferential wall thickening of the esophagus. CECT, contrast-enhanced computed tomography

**Figure 2:**
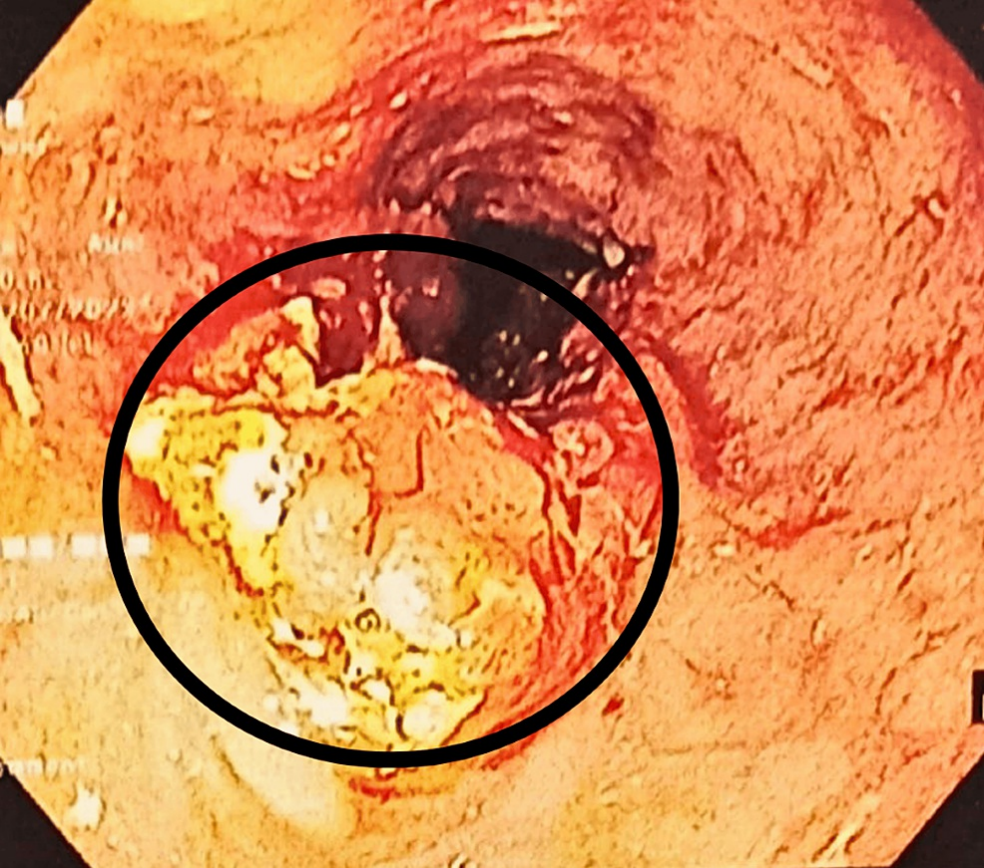
Upper GI endoscopy The circle shows semi-circumferential ulceroproliferative friable growth.

The patient underwent a clinical and radiological examination. Contrast-enhanced computed tomography (CECT) thorax with abdomen and pelvis demonstrated asymmetrical circumferential wall thickening of the esophagus over a length of approximately 7.5 cm (D5-D7 level) with a maximum thickness of 16 mm. The involvement of necrotic lymphadenopathy, as described above, was suggestive of malignant etiology. Patchy round glass opacities and centrilobular nodules with a tree-in-bud pattern in the right lung and cystic bronchiectatic changes in the left upper lobe were seen (Figure 1). Upper GI endoscopy showed a hemicircumferential ulceroproliferative friable growth at 22-28 cm from the central incisors, causing luminal compromise (Figure 2).

Pre-chemotherapy physiotherapy intervention

The patient underwent a physiotherapy intervention before chemotherapy for a duration of eight days (Table 1).

**Table 1:** Physiotherapy intervention TEE, thoracic expansion exercises; TENS, transcutaneous electrical nerve stimulation

Goals	Intervention	Rehabilitation program
Patient education	Guidance to the patient and the patient’s caregivers	The condition of the patient was explained, along with the benefits and significance of physiotherapy. The patient and her family were provided with comprehensive explanations detailing how the prescribed treatment would ameliorate her health status by mitigating potential complications, consequently diminishing breathlessness and fatigue levels, and enhancing the strength of both upper limbs and lower limbs
To relieve breathlessness	Dyspnea-relieving position in side lying, sitting, and standing	Every time the patient became dyspneic, she was asked to perform dyspnea-relieving positions
To improve swallowing	Shaker exercise and Mendelsohn maneuver	10 reps × three sets; progression: 20 reps × three sets
To enhance pulmonary ventilation and oxygenation to prevent fatigue	Diaphragmatic breathing exercises and TEE	10 reps × one set, thrice a day
To improve the strength of the upper and lower extremities	Strengthening exercises	Using weight cuffs (10 reps × two sets, thrice a day)
For core muscle strengthening	Pelvic bridging (single and double leg), static glutes, static adductors, and abductors	10 reps × one set, thrice a day, with a 10-second hold (starting from day 2)
For increasing functional capacity and fitness	Aerobic exercises and bedside walking	10 minutes; progression after day 2: 20 minutes
To reduce anxiety and promote relaxation	Jacobson’s relaxation technique	15 minutes, twice a day
To relieve cancer-related pain	Low TENS	10-15 minutes

The post-chemotherapy intervention is summarized in Table 2.

**Table 2:** Post-chemotherapy intervention TEE, thoracic expansion exercises; TENS, transcutaneous electrical nerve stimulation

Goals	Intervention	Rehabilitation program
Patient education	Guidance to the patient and the patient’s caregivers	The condition of the patient was explained, along with the benefits and significance of physiotherapy. The patient and her family were provided with comprehensive explanations detailing how the prescribed treatment would ameliorate her health status by mitigating potential complications, consequently diminishing breathlessness and fatigue levels, and enhancing the strength of both upper limbs and lower limbs
To relieve breathlessness	Dyspnea-relieving position in side lying, sitting, and standing	Every time the patient became dyspneic, she was asked to perform dyspnea-relieving positions
To improve swallowing	Shaker exercise and Mendelsohn maneuver	10 reps × three sets; progression: 20 reps × three sets
To relieve fatigue	Energy conservation techniques and pacing exercises	-
To enhance pulmonary ventilation and oxygenation to prevent fatigue	Diaphragmatic breathing exercises and TEE	10 reps × one set, thrice a day
To improve chest expansion	TEE (anterior and lateral)	10 reps × one set, thrice a day
To improve the strength of the upper and lower extremities	Strengthening exercises	Using weight cuffs (10 reps × two sets, thrice a day)
For core muscle strengthening	Pelvic bridging (single and double leg), static glutes, static adductors, and abductors	10 reps × one set, thrice a day with a 10-second hold (holds starting from day 2)
To relieve nausea and vomiting	Aromatherapy with lavender and rosemary oil	10 minutes
For increasing functional capacity and fitness	Aerobic exercises and bedside walking	10 reps × one set, thrice a day, with a 10-second hold (starting from day 2)
To reduce anxiety and promote relaxation	Jacobson’s relaxation technique	15 minutes, twice a day
To relieve cancer-related pain	Low TENS	10-15 minutes

The same protocol will be followed after each cycle of chemotherapy. If the patient finds trouble doing the exercise, the number of sets is reduced, and the pacing time is increased.

Follow-up and outcome measures

A follow-up was carried out after three months of therapeutic treatment. After the rehabilitation program, upper and lower limb muscle strength increased from 3/5 to 4/5 on both sides. Outcome measures included Functional Assessment of Cancer Therapy-Esophagus (FACT-E), Fatigue Severity Scale and mMRC grading of dyspnea, Numerical Pain Rating Scale (NPRS), and Eating Assessment Tool 10 (EAT-10) (Table 3).

**Table 3:** Outcome measures Grade 1: I get short of breath when hurrying on level ground or walking up a slight hill; Grade 2: On level ground, I walk slower than people of the same age because of breathlessness or have to stop for breath when walking at my pace EAT-10, Eating Assessment Tool 10; FACT-E, Functional Assessment of Cancer Therapy-Esophagus; mMRC, modified Medical Research Council; NPRS, Numerical Pain Rating Scale

Outcome measures	Pre-intervention	Post-intervention
FACT-E	66	46
Fatigue Severity Scale	48	28
EAT-10	34/40	27/40
mMRC grading	Grade 2	Grade 1
NPRS	On activity: 4/10; on rest: 3/10	On activity: 2/10; on rest: 1/10

## Discussion

Esophageal cancer and its curative treatment, including neoadjuvant chemotherapy, exert a notable influence on the physical fitness of affected individuals. Neoadjuvant chemotherapy for esophageal cancer patients often comes with side effects that affect patients’ appetite and body weight and lead to a marked decrease in physical activity that may reduce strength, muscle mass, and functional walking capacity. It is important to evaluate the functional effects of weight and muscle loss, which are acknowledged side effects of esophageal cancer. Due to the absence of distinctive clinical symptoms in early-stage esophageal cancer, a majority of individuals who fail to undergo early diagnosis are frequently identified at an advanced disease stage, leading to diminished QOL and an unfavorable prognosis [14]. Chemotherapy affects multiple systems, such as cardiovascular, respiratory, musculoskeletal, gastrointestinal, and hematopoietic systems, and has a significant impact on the patient’s overall QOL [15]. A limited number of research studies have investigated the beneficial effects of physical rehabilitation in esophageal cancer before and after chemotherapy for dealing with the side effects of the chemotherapy and disease.

A study by Christodoulidis et al. suggested that pre-rehabilitation in patients undergoing surgery showed better results post-surgery [16]. In another study conducted by Inoue et al., patients receiving preoperative respiratory rehabilitation, including deep diaphragmatic breathing and muscle strength exercises for upper and lower limbs and abdominal muscles, reported a considerably decreased incidence of postoperative pulmonary complications [17]. Similarly, we gave strength training to the upper and lower limbs and respiratory rehabilitation to our patients and found a positive impact. Strength and exercise training aided in the management of this esophageal cancer patient undergoing chemotherapy by helping to mitigate treatment-related side effects and improve overall physical function. These exercises enhanced muscle strength and endurance, which alleviated chemotherapy-induced fatigue, maintained mobility, and supported the body’s ability to tolerate treatment.

The objectives of dysphagia management and rehabilitative interventions encompass the prevention of malnutrition, dehydration, and pneumonia, alongside the normalization of diagnosed physiologic swallow dysfunction, ultimately enhancing the patient’s QOL [18]. A study by Ohba et al. concluded that Shaker exercise helped preserve swallowing functions in patients with head and neck cancer. We also incorporated a Shaker exercise, which improved swallowing [19].

A tailored physical therapy program integrated into comprehensive palliative care enhanced mental well-being, health-related QOL, and the stress level of carers for patients with severe chronic illnesses and cancer [20,21]. In the case described above, rehabilitation was administered to an individual diagnosed with esophageal cancer, manifesting secondary complications attributed to chemotherapy, including generalized weakness, dyspnea, nausea and vomiting, dysphagia, and heightened anxiety. Our treatment protocol helped with fatigue management through energy conservation techniques and pacing activities, muscle weakness by strengthening exercises, relaxation techniques to improve overall comfort, breathing exercises to maintain lung function, improved swallowing coordination, and nausea and vomiting. Strength as well as breathing exercises are contributors to recovery, but they are not the sole factors. Other components, such as medications and therapeutic interventions like Shaker exercise, etc., played a role in the overall recovery process.

However, while integrating physiotherapy into the treatment of these patients, there are a few possible drawbacks or challenges to take into account. Patients undergoing chemotherapy may have significant fatigue, which may be made worse by adding physical therapy to their treatment plan. Exercise regimen duration and intensity must be carefully adjusted to the individual’s overall condition and energy level. Careful planning of physiotherapy sessions is necessary to reduce the chance of infections or accidental harm during physical therapy sessions.

Further research is necessary to explore these findings thoroughly, considering each factor that may affect the result in a large, standardized group of patients. For instance, exploring additional research into the toxicity of chemotherapy may be warranted.

## Conclusions

Palliative care was the main focus of this case study’s intervention. Rehabilitative interventions within the realm of physiotherapy have demonstrated notable efficacy for individuals undergoing chemotherapy. This case study reveals that well-planned physiotherapeutic interventions given pre- and post-chemotherapy showed improved treatment outcomes and were endorsed to be incredibly beneficial in relieving breathlessness, reducing fatigue, and enhancing muscle strength, QOL, and functional independence in a patient with carcinoma of the esophagus.
